# Intercepting moving targets: does the visuomotor latency depend on whether one taps on the target or slides through it?

**DOI:** 10.1007/s00221-026-07264-3

**Published:** 2026-03-16

**Authors:** Eli Brenner, Sem Bom, Jeroen B. J. Smeets

**Affiliations:** Department of Human Movement Sciences, Vrije Universiteit Amsterdam, 1081 BT Amsterdam, Netherlands

**Keywords:** Interception, Online control, Goal-directed movements, Latency, Human

## Abstract

When trying to intercept a moving target, one’s movements are continuously adjusted to match the latest information about the target’s position. Many target characteristics influence the latency of such adjustments. Does the kind of movement that is being made also influence this visuomotor latency? Since there are reasons to suspect that it might, we compared the latency of responses to sudden jumps in moving targets’ positions when making two quite different movements to intercept the targets: sliding one’s finger across a screen to pass through the moving targets and lifting one’s finger off the screen to tap on the targets. Twenty-two participants intercepted targets by making both these movements in two separate blocks of trials in counterbalanced order. Despite the substantial differences between the two kinds of movements, the latency of the responses was 114 ms for both. Thus, the visuomotor latency does not depend on the kind of movement that is made.

## Introduction

Human movements are continuously guided by the latest available visual information (Brenner and Smeets 2025). There is a long tradition of studying this by examining how ongoing goal-directed arm movements are adjusted if the target’s position suddenly changes (Pélisson et al. 1986; Prablanc and Martin 1992; Soechting and Lacquaniti 1983). Studies have reported a wide range of values for the latency for making such adjustments (the visuomotor latency). Some of the variability in the reported values arises from differences in how the latency was determined (Oostwoud Wijdenes et al. 2014), rather than from actual differences in visuomotor latency. But actual differences in visuomotor latency do arise from visual properties of the target, such as how the target differs from the background and from other items (Kozak et al. 2019; Veerman et al. 2008), because how quickly visual information is processed depends on such properties. For instance, visual processing is slower when the contrast is low (Groen et al. 2022; Oram 2010) or when target identification depends on fine detail (Hegdé, 2008). Actual differences in visuomotor latency also arise from different muscles being involved in the motor response. The time it takes to activate muscles in response to visual information depends on the distance that signals must travel to do so (in terms of nerve lengths) and on the conduction velocity of the nerves involved (Crevecoeur and Kurtzer 2018; Ingram et al. 1987; Zhang et al. 2020).

Beside depending on how the visuomotor latency is determined, on how long the visual processing takes, and on the muscles involved in the response, the visuomotor latency has also been reported to depend on various other factors. One such factor is how directly the required response is related to the change in the visual information: the visuomotor latency is shorter for an intuitive response such as adjusting the movement to reach the target, than for responses that are a consequence of specific instructions such as to move in the opposite direction than the direction in which the target moved (Day and Lyon 2000) or to divert the movement toward a target indicated by a spatial or symbolic cue if one suddenly appears (de Brouwer and Spering 2022). A second factor is the visibility of the hand: the visuomotor latency is shorter if the hand is visible than if it is not (Reichenbach et al. 2009). A third factor is how the task is framed: the latency is shorter for a target displacement than for the identical displacement of a gap between obstacles (Aivar et al. 2015). We wondered whether the visuomotor latency also depends on the movement itself: whether it would be different for two different movements if we ensured that they made use of identical visual information and if the response was likely to rely on the same muscles.

To answer this question, we compare two kinds of movements that have been reported to differ in visuomotor control: sliding through a target and tapping on a target. It has been claimed that sliding along a surface to reach a target relies more heavily on visual feedback than does moving off the surface to tap on the target (Chaston et al. 2024). It has also been suggested that movement endpoints are represented fundamentally differently than positions that one is asked to move through on the way to the endpoint (Ghez et al. 2007; Scheidt and Ghez 2007). Given these differences, the visuomotor latency might also be different for the two kinds of movements. Indeed, comparing the figures of studies in which participants made sliding movements (e.g. Aivar et al. 2015; Brenner and Smeets 2023; de Brouwer and Spering 2022; Numasawa et al. 2022) with the figures of studies in which participants tapped on targets (e.g. Brenner et al. 2023; Brenner and Smeets 2015) suggests that the visuomotor latency is at least 10 ms longer when making sliding movements. But there were many differences between the studies. Different methods were used to measure and analyse the movements, the movements had different extents, the targets had different sizes, colours, and contrasts with the background, the orientation of the surfaces across which the movements were made was different, as was whether participants saw their actual hand or a cursor that followed the hand (with some delay). Moreover, participants were seated in the studies in which they made sliding movements, while they were standing in the tapping studies. Here, we compare the visuomotor latency for sliding and tapping movements using the same equipment, stimuli and analysis for both kinds of movements.

## Methods

### Participants and design

20 healthy, naïve participants (18–30 years old) and two of the authors (26 and 67 years old) took part in the experiment. The procedure, including naïve participants signing an informed consent form, was in accordance with our ethical approval. Each participant performed a block of trials in which they had to slide through the target and a separate block of trials in which they had to tap on the target. The order of the blocks was counterbalanced across participants.

### Stimuli and equipment

**Fig. 1:**
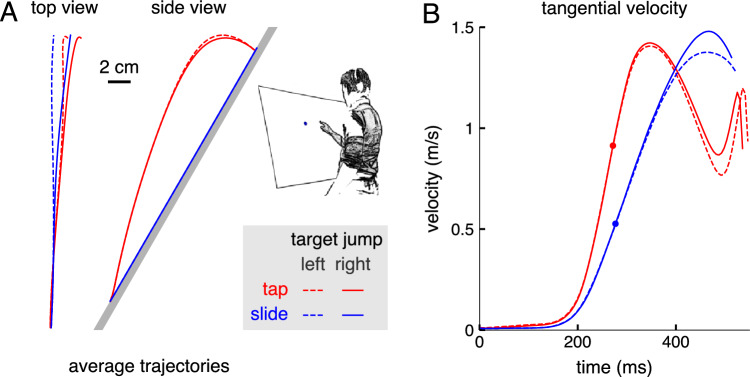
Average trajectories for the two kinds of movements (colours) and directions of target jumps (line styles). **A**. Average finger paths parallel to the surface (top view) and as seen from the side (side view; the screen is represented by the grey bar). The inset shows an impression of a participant tapping on a target. **B**. Average tangential velocity as a function of the time from when the moving target appeared. The dots indicate the average moments of target jumps in the corresponding trials

We used a setup in which participants stood in front of a large screen (as in Brenner et al. 2023) and tried to intercept moving targets that were projected from behind onto that screen. They did so both with sliding movements (as in Brenner and Smeets 2023) and with tapping movements (as in Brenner et al. 2023). The screen (Techplex 150; 125 × 100 cm) was tilted backward by 30° (see picture in Fig 1A). The target and starting point, as well as the white background, were projected onto that screen using an In-Focus DepthQ Projector (800 × 600 pixels; 120 Hz). The target was a 2 cm diameter blue disk that moved rightward at 60 cm/s from a position 30 cm above and 30 cm to the left of the starting point. The starting point was a 2 cm diameter green disk that disappeared as soon as the target appeared and reappeared as soon as the target disappeared, so at any moment only one of the two disks was visible.

The position of the tip of the index finger of the participant’s dominant hand was determined at 500 Hz using an Optotrak 3020 system that measures the positions of infra-red markers. One such marker was attached to the nail of the participant’s index finger. The position of the images on the screen with respect to the tip of this finger was determined using a calibration procedure whereby the participant placed the tip of their finger on four points on the screen. Precise synchronisation of the measured finger positions with respect to the timing of the images was achieved by briefly interrupting the signal to a second Optotrak marker whenever a new target was presented. On the first frame with a new target, and when the target jumped, a flash was presented at the top left corner of the screen. This flash illuminated a light-sensitive sensor. The signal that resulted from the increase in light falling on the sensor briefly turned the second marker off. In this way, the appearance of the target and the moment the jump was visible on the screen were detected in synchrony with the measured finger positions.

### Procedure

For the block of trials with tapping movements, participants were instructed that they had to lift their finger off the screen and tap on the target, without specifying how far the finger should move from the screen. For the block of trials with sliding movements, they were instructed to slide their finger across the screen without losing contact with the screen, and to hit the target in a manner that would push it away if it were a real disk. They were not asked to hit the target quickly, but they obviously had to do so before the target left the screen. Based on earlier studies (e.g. Brenner et al. 2023) we expected participants to try to hit the targets after about 500 ms, so when the target was laterally aligned with the starting point. This would have the advantage that the main movement direction hardly has a lateral component, making it easier to isolate the responses to the lateral target jumps.

A trial started when the participant placed his or her fingertip on the starting point. After a random interval of between 600 and 1200 ms from when the trial started, the target appeared. If the fingertip left the starting point before the target appeared, the trial was aborted and had to be started again by placing the fingertip on the starting point again. Once the target appeared, participants could move to try to hit it. Not only how they had to move differed between the blocks (tap or slide), but also the feedback that they received.

For the tapping movements, taps were recognised by the finger’s deceleration when it hit the screen (threshold of 50 cm/s^2^ for acceleration orthogonal to the screen as determined from three consecutive samples). If the finger hit the screen within the bounds of the target, the target stopped where it had been at the time of the hit. If the finger hit the screen outside the target, the target moved away from the finger (along a line connecting the position of the tap with where the target was at the time of the tap). If the finger hit the screen too gently, the target simply continued moving.

For the sliding movements, whether the finger had hit the target was determined continuously by considering the positions of the finger and target (interpolating between measured finger positions and rendered target positions). As soon as the finger touched the target, the target was considered to have been hit, and it moved away in the direction in which it had been hit. If the finger missed the target, the target simply continued to move as it was moving before. In addition to the different kinds of visual feedback that were presented, a successful hit was indicated by a sound. The sound was the same for both kinds of movements.

For each kind of movement, participants performed a block of 200 trials. In each trial, the target jumped 1 cm laterally, randomly either to the left or to the right, but ensuring that it jumped to the left on 100 trials and to the right on the other 100 trials. The jump was triggered when the finger moved beyond the extent of the (no longer visible) starting point in the plane of the screen. Due to measurement and rendering delays, the jump affected the target position on the screen about 33 ms later. At that moment, the jump was added to the ongoing rightward motion of the target. Participants performed 10 practice trials to get accustomed to the kind of movement they were required to make before each block of trials. The target did not jump in the practice trials.

### Analysis

For the tapping movements, there were occasional trials in which participants tapped too gently. For those trials, taps were later identified as any moment at which the finger was less than 5 mm from the screen and stopped moving towards the screen. For the sliding movements, there was no obvious end of the movement when the finger missed the target. In that case, we considered the moment that the finger crossed the target centre’s path to be the end of the movement. When the finger hit the target, the end of the movement was the moment of the hit, although the finger obviously continued moving after it hit the target.

To provide an overall impression of the two kinds of movements, we determined the fingers’ average movement trajectories and tangential velocities. We considered all trials in which the marker on the finger was visible throughout the movement. To average across trials of different durations, the position and velocity of the finger was estimated for 100 equal steps in the time between when the target appeared and the end of the movement (so for 101 equally spaced time points). We used a Savitzky-Golay filter (based on a second-order polynomial and a 20 ms time window) to simultaneously interpolate, differentiate (for determining the velocity), and slightly smooth the data. The 101 positions in each of the three directions, and the 101 tangential velocities derived by combining the velocities in the three directions, were then each averaged across trials. This was done separately for each kind of movement and direction of the target jump, first within participants and then across participants. The tangential velocities were plotted as a function of ‘actual’ time by scaling the relative time, defined by the 101 time points, by the mean time in the included trials between when the target appeared and the end of the movement.

To check whether the overall performance and the timing of the movements was similar for the two kinds of movements, we determined the percentage of the targets that were hit, as well as the median time between when the target appeared and the end of the movement. This was done for each block of each participant, considering all the trials.

For our main measure, the visuomotor latency, we determined the response to the target jumps. This is the difference between the lateral velocity after leftward and rightward target jumps, such that a positive difference corresponds with a response in the direction of the jump. Relying on the difference isolates the response to the target jump from any other lateral velocity of the finger. For this analysis, we again only considered trials in which the marker on the index finger remained visible throughout the movement, but we also only considered trials in which the finger took at least 200 ms from when the target jumped to hit the screen (when tapping) or to hit or pass the target (when sliding). This allowed us to average the difference in lateral velocity for 200 ms from when the target jumped, without any extrapolation, interpolation or smoothing. The latency of the response was determined following the method proposed in Oostwoud Wijdenes et al. (2014). This involves first averaging the response across participants and then extrapolating a line through the points at which the response reached 25% and 75% of its peak value back to the point at which the difference in velocity is zero. Considering this point to be the onset of the response, the latency is the time from when the target jumps until this point. To also obtain a measure of the precision of the estimates of the latency, we determined the latency in this manner using 10,000 bootstrapped samples for each kind of movement. Each of the samples was based on a random selection of participants, with for each participant a random selection from that participant’s trials for each direction of the target jump. The random selections (random sampling with replacement) had the same number of participants as the actual number of participants, and the same number of trials as the number of measurements for each direction of the target jump for the participant in question. We report the mean and standard deviation of the latencies for each kind of movement. The standard deviation provides a measure of the confidence in the mean.

To assess whether participants adjusted their movements differently in the two blocks, we determined to what extent participants adjusted the path and the timing of their movements to the target jumps. For each participant, we determined how much further to the right the movements ended in trials in which the target jumped to the right than in trials in which it jumped to the left. The difference between the median lateral positions is a measure of the extent to which the path was adjusted. Dividing this difference by 2 cm (the distance between where the target would be after jumping to the left or to the right) gives the fraction of the jump that is compensated for by changing the position towards which the finger moves. Similarly, we determined how much sooner the movements ended, with respect to the moment the target appeared, when the target jumped to the right than when it jumped to the left. The difference between the median times taken is a measure of the extent to which the timing was adjusted. Dividing this difference by 33.3 ms (the time it takes the target to move the 2 cm between target positions after leftward and rightward jumps) gives the fraction of the jump that is compensated for by changing how fast the finger moves. For this analysis, we could again consider all trials in which the marker on the finger was visible throughout the movement.

The data and python script for analysing the data can be found at: https://osf.io/e9rhf/overview

## Results

**Fig. 2:**
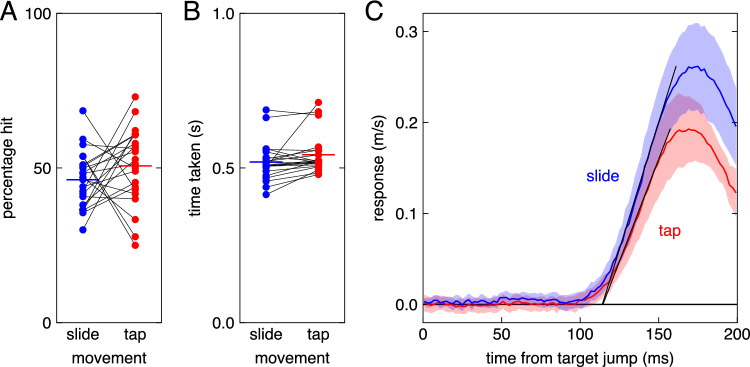
Performance and response. **A**. The percentage of targets that were hit. **B.** The median time taken to hit or miss the target. In both panels, values for the same participant are connected by thin lines, and mean values are indicated by coloured, thick horizontal lines. **C**. The response to the target jumps (the difference between the average lateral velocity of the finger after jumps to the left and after jumps to the right) as a function of the time after the jump. The curves show the mean responses for the two kinds of movements. The shaded areas indicate the 95% confidence intervals across participants. The latency is about 114 ms for both kinds of movements: where the thin lines (determined as explained in the “Analysis” section) intersect the time axis

We could include 8773 of the 8800 trials (99.7%) when determining the trajectories. An obvious difference between the trajectories when performing the two kinds of movements was that the finger left the screen to tap on the target but did not leave the screen when sliding to the target (Fig. 1A). When tapping, the average maximal distance between the finger and the screen was 5.5 cm (ranging from 4.4 to 8.3 cm across participants). When tapping, the target jumped about 272 ms after the target appeared (mean across participants of the median value per participant). When sliding, the hand initially moved more slowly, so the target jumped 5 ms later (Fig. 1B). For sliding movements, the finger reached the target just after reaching its maximal velocity. For tapping movements, there are two peaks in velocity. The trough that separates the two peaks occurs when the fingertip’s path curves (Viviani and Terzuolo 1982). The path curves as the finger moves towards the screen to tap on the target, after first moving up (and slightly away from) the screen to a suitable position from which to tap (Fig. 1A). Thus, the two kinds of movements had quite different kinematics. But despite the considerable difference in kinematics, overall performance was quite similar for both kinds of movements (Fig. 2A, B). The percentages of targets hit were 46 ± 4 and 51 ± 5 for the sliding and tapping movements, respectively. The times taken were 520 ± 26 ms and 543 ± 26 ms (means with 95% confidence intervals).

Having established that there are clear differences between the two kinds of movements, we can now turn to our main measure: the visuomotor latency. For the analysis of the visuomotor latency, we could include 8329 of the 8800 trials (almost 95%). The response was larger for sliding movements, but the latency was about 114 ms for both kinds of movements (Fig. 2C). With the help of bootstrapping, we obtained estimates of 114.4 ± 2.4 ms for the latency when tapping, and 114.1 ± 2.3 ms for the latency when sliding (means ± standard deviations of the bootstrapped latencies). Thus, we find no evidence for a difference in latency between the two kinds of movements.

**Fig. 3:**
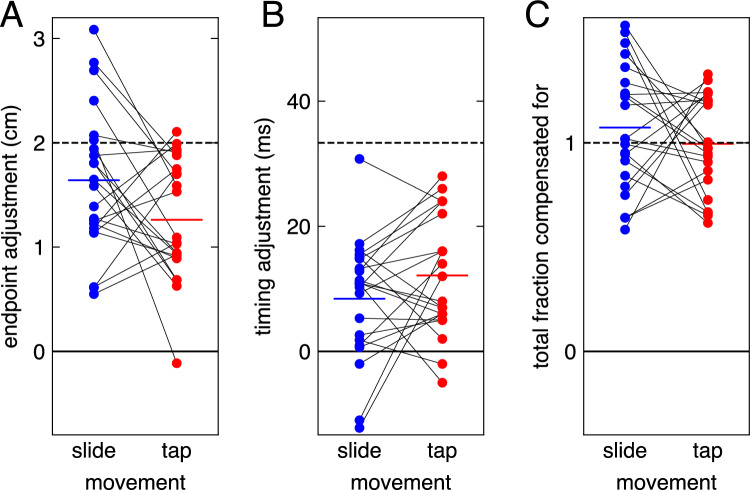
The extent to which participants compensated for target jumps. The three panels indicate the compensation realised by adjusting the finger’s path (**A**), its speed (**B**), and the total compensation (**C**). Dots indicate individual participants’ values (thin lines connect values for the same participant). Coloured, thick horizontal lines indicate the mean across participants. The black dashed horizontal lines indicate the adjustment that is required to fully compensate for the target jumps

That the latency of adjustments was the same for both kinds of movements might give the impression that the response to target jumps was altogether identical in both cases. But it was not: the response was weaker for tapping movements than for sliding movements (Fig. 2C). This response measure only captures spatial adjustments, but participants might also have adjusted their timing (Bertonati et al. 2025). On average, *sliding* movements ended 1.64 ± 0.28 cm further to the right and 8.4 ± 3.9 ms earlier when the target jumped to the right than when it jumped to the left (Fig. 3; for this analysis we could again consider 99.7% of the trials; the values are averages across participants with their 95% confidence intervals). This means that for sliding movements, on average, participants compensated for 82% of the target jump by adjusting their finger’s path and 25% of the target jump by adjusting their timing (together, on average 107 ± 12%). On average, *tapping* movements ended 1.26 ± 0.24 cm further to the right and 12.1 ± 3.9 ms earlier when the target jumped to the right than when it jumped to the left. This means that on average, participants compensated for 63% of the target jump by adjusting their finger’s path and 36% of the target jump by adjusting their timing (together 99 ± 10%). The average difference of 0.38 cm between the movement endpoints (Fig. 3A) has a 95% confidence interval of 0.03 cm to 0.73 cm when comparing individual participants’ values across the two kinds of movements, so we can be quite confident that participants adjusted the position of the finger more when sliding than when tapping. Thus, not only the overall kinematics, but also the response to perturbations was different for the two kinds of movements. We cannot be confident that participants adjusted the timing of their movement more when tapping than when sliding, because the average difference of 3.7 ms has a confidence interval of -0.8 to 8.2 ms.

## Discussion

We examined whether the visuomotor latency of lateral adjustments to arm movements was different when trying to tap on targets than when trying to slide through identical targets. We found no difference in visuomotor latency between the two kinds of movements (Fig. 2C). In both cases, the visuomotor latency was about 114 ms, which is similar to the latency that we found in previous studies that used this setup (Brenner et al. 2023; Brenner and Smeets 2015). Thus, the visuomotor latency does not depend on whether one taps on the target or slides through it.

Of course, the visuomotor latency might differ between other arm movements, especially if different visual information or different muscles are involved, or if the goals of the movements differ. The goal of both our sliding and tapping movements was to intercept moving targets. Moreover, the targets were identical in both blocks of trials, as were the starting points, so some characteristics of the movements were similar in both blocks of trials. But there were also clear differences. Besides the obvious difference in the finger’s distance from the screen (see side view in Fig. 1A), we also found clear differences between the velocity profiles (Fig. 1B). Moreover, we found that participants adjusted the two movements differently: they relied more on adjusting the finger’s position when sliding than when tapping (Fig. 2C and Fig. 3A). We intentionally did not choose movements that also differed from each other in many other aspects than these, because we wanted the same muscles to be used to respond to the target jump for both kinds of movements. Of course, we do not know whether this is indeed the case, but it seems likely that the same muscles were used, because despite the movements themselves involving different muscle activation patterns, the arm was in a similar posture when responding to the same change in target position. Thus, this study demonstrates that for a given change in the stimulus and approximate arm posture, the latency for responding to the change does not depend on the velocity profile and whether the finger remains in contact with the surface.

### Possible limitations

A methodological issue that arises when comparing the two kinds of movements is that a consequence of the finger stopping at the target when tapping, but not when sliding, is that the movement endpoint is determined differently for the two kinds of movements. For both kinds of movements, we considered the moment that determined the feedback that was provided to our participants to be the movement endpoint, because we know that people adapt their behaviour to such feedback within a few trials (Brenner et al. 2023). But this leaves some ambiguity in the choice of an endpoint for movements that slide past the target, missing it, because in that case the target just continues moving, so there is no explicit feedback about when and where the target was missed. We took the moment the finger clearly passed the target to be the endpoint. Since participants hit the near edge of the target when sliding their finger to the target, and they received more informative feedback when they hit the target than when they missed it, they might have aimed to complete the adjustment to the target jump at the average time and place at which they hit such targets, without considering when and where they missed targets. This would be when the finger had moved up to 1 cm less far from the starting point than we now consider to be the movement endpoint. If so, the end of the movement could be up to 8 ms earlier, because the finger was moving at about 1.3 m/s (Fig. 1B) orthogonally to the target motion (Fig. 1A).

Relying on this earlier endpoint would not affect measures that are independent of the definition of the endpoint, such as the observed differences between the trajectories (Fig. 1) or between the responses (Fig. 2C). We were mainly interested in the visuomotor latencies, that are estimated from the responses and are therefore independent of the definition of the endpoint. The only way in which the estimated visuomotor latency could be affected by how the movement endpoint is determined is through how the endpoint influences which trials are excluded from the analysis of the latency. Considering the endpoint of sliding movements to be when the finger is aligned with the closest edge of the target, rather than when it is aligned with the centre of the target, does not change the number of excluded trials. We can therefore be certain that the definition of the endpoint did not influence our estimate of the visuomotor latency.

Considering an earlier endpoint for sliding movements would influence the distribution of adjustments across space and time (Fig. 3). The estimates of the adjustment to the target jump would be slightly smaller, both in terms of the target’s path (Fig. 1A; the blue curves in the top view diverge) and in terms of the timing of the movement endpoint (Fig. 1B; the separation between the solid and dashed blue curves shows that the difference in speed, and therefore the time difference, builds up during the last 100 ms of the movement). Participants having considered an earlier movement endpoint for the sliding movements would therefore explain why, on average, our analysis suggested that participants overcompensated for the jump when making sliding movements (combined adjustment of 107%). Although considering an earlier movement endpoint and therefore less compensation for sliding movements would slightly reduce the difference between the two kinds of movements in terms of the adjustment to the path, it would increase the difference in terms of adjustments to the timing, so we would be less certain of the larger adjustment to the endpoint when sliding, but more certain of the smaller adjustment to the timing.

It is understandable that the response was more vigorous for sliding movements than for tapping movements (Fig. 2C): there was less time to adjust the movement because the target jumped later and the finger reached the target earlier. The target jumped about 5 ms later, because although the finger started moving at about the same time for both tasks, it initially moved less fast for sliding movements (Fig. 1B). The movement ended earlier, because there was no need to slow down near the end. But why did participants not only adjust their sliding movements more vigorously, but also rely more on changing the endpoint than the timing (Fig. 3A and B)? We suspect that this somehow reflects differences in how one can best adjust the two kinds of movements (Liu and Todorov 2007). Maybe participants rely more on adjusting the timing when they receive better feedback about their timing. For both kinds of movements, participants could always see both their hand and the target. But when tapping, participants also received haptic feedback about when the movement ended: they felt their finger hit the screen. This was even so on the occasional trials in which participants tapped too gently. Moreover, when they missed the target, the target moved away from where they tapped (see “Procedure” section of the “Methods”), indicating whether they had tapped ahead of or behind the target. And when they hit the target, they received feedback about their timing in terms of the lateral position of the target beneath their finger, because the target remained visible for 500 ms at its position at the time of the tap. When sliding, participants received some feedback about how they had hit the target when they did so, from which they could infer when they had done so to some extent, but they received no haptic feedback about the timing of the hit, and they received no explicit feedback if they missed the target.

We included the data of two of the authors in our analysis. We did so because we considered it to be unlikely that people could intentionally change the latency of the response, since it seems to be quite automatic (Day and Lyon 2000; Pisella et al. 2000). Nevertheless, we made sure that this did not affect our conclusions by also determining the latency if the authors’ data is excluded. In that case, the average latency is slightly shorter (113.8 ± 2.5 ms for tapping; 112.8 ± 2.2 ms for sliding), presumably because on average the authors were older than the naïve participants, and the visuomotor latency increases with age (Kimura et al. 2015), at least partly because motor units’ conduction velocities decrease with age (Wang et al. 1999). Although we did not expect the visuomotor latency to change with practice, we counterbalanced the two kinds of blocks. We checked that the order does not matter by comparing the first and second (last) block, irrespective of the kind of movement. The average latency clearly did not decrease with practice, because it was 113.5 ms for the first block and 114.4 ms for the second block. On average, participants hit 47% of the targets in the first block, and 50% in the second block. The average times taken were 535 and 528 ms for the first and second block, respectively.

## Conclusion

We found that the latency of responses to target jumps is the same when tapping on a moving target as when sliding through such a target. Thus, although the latency with which arm movements respond to new visual information depends on the visual information and the muscles used for the response, it does not appear to depend on details of the movements that the brain has to consider when transforming the visual input into the motor commands that lead to an appropriate response.

## Data Availability

The data and python script for analysing the data can be found at: https://osf.io/e9rhf/overview.
